# Prevalence and risk factors of elder abuse in survivors of stroke: A cross‐sectional study

**DOI:** 10.1002/hsr2.1616

**Published:** 2023-10-13

**Authors:** Guifen Zhang, Minyuan Chen, Peiyi Xiao, Zhibo Peng

**Affiliations:** ^1^ Department of Emergency Shunde Hospital, Southern Medical University (The First People's Hospital of Shunde) Foshan Guangdong China

**Keywords:** elder abuse, insomnia, prevalence rate, risk factors, stroke survivors

## Abstract

**Background and Aims:**

The understanding of the prevalence and risk factors associated with elder abuse in stroke survivors is currently lacking. Therefore, the objective of this study is to ascertain the prevalence and potential risk factors of elder abuse in stroke survivors, while also examining its correlation with insomnia.

**Methods:**

In this cross‐sectional study, a total of 485 stroke survivors aged 65 years and older, who received treatment at the Emergency department of Shunde Hospital, Southern Medical University, were subjected to face‐to‐face interviews using the questionnaire on elder abuse from the Third Survey on Chinese Women's Social Status. A logistic regression analysis was employed to examine the association between risk factors and elder abuse among stroke survivors.

**Results:**

62.27% of the participants reported experiencing elder abuse, with 14.85% of them indicating suffering from more than two subtypes of abuse. Factors such as residing in nursing homes, lower income, and smoking were found to increase the likelihood of experiencing elder abuse and all four subtypes of abuse. Additionally, advancing age was associated with a higher risk of experiencing all four subtypes of abuse, although it did not affect the occurrence of overall abuse. It is worth noting that the self‐reported prevalence of the four types of abuse by the elderly themselves was higher compared to the reports provided by caregivers.

**Conclusion:**

Elder abuse is prevalent among stroke survivors, especially those who are residing in nursing homes, with lower income, and smokers. Elder abuse significantly increased the prevalence of insomnia in stroke survivors. Further research is needed to better explore effective measures to reduce the prevalence of elder abuse of stroke survivors.

## 1 INTRODUCTION

China possesses one of the highest aging rates globally, with a steadily increasing elderly population that has garnered significant attention.^1^ The number of individuals over 60 in China rose from 222 million in 2015 to 264 million in 2021.^2^, ^3^ Projections indicate that by 2025, the elderly population in China will reach 280 million by 2025, accounting for 20% of the total population. This aging population has contributed to a substantial increase in stroke prevalence, with an annual growth rate of 8.7%.^4^ Statistics reveal that more than half of the world's new stroke cases occur in China each year, affecting around 5.5 million individuals.^5^, ^6^ As stroke prevalence continues to rise and medical advancements improve in China, the stroke mortality rate has declined.^7^ Consequently, the population of stroke survivors is expected to significantly increase in the future. Stroke patients often face cognitive and physical impairments, leading to a loss of independence in daily activities, and requiring extensive and time‐consuming care. Nevertheless, the growing responsibility of caring for stroke survivors often presents challenges.

Elder abuse refers to the harm or distress inflicted upon elderly individuals through single or repetitive actions, or the failure to fulfill expected responsibilities within a fiduciary relationship.^8^ This abuse is a major contributor to a decline in the quality of life, poorer health outcomes, and increased medical burdens among the elderly. It can take in various forms, including physical, emotional, sexual, and economic abuse.^9^ Neglect, which is another form of elder abuse, occurs when caregivers fail to meet the basic needs of the elderly, and it often remains hidden. The global prevalence of elder abuse is 15.7%, but it varies significantly across different countries.^10^ In China, the third survey of women's social status reveals a prevalence of elder abuse at 13.3%, with emotional abuse being the most common type.^11^ Elder abuse prevalence varies between rural and urban areas, with an overall rate of 16.2% in rural areas, which is nearly double that of urban areas.^12^ A survey conducted at a prominent medical center in Nanjing, China, involving 412 participants aged 60 or above, discovered that 35% of the subjects had experienced elder abuse.^13^ Recent studies indicate that elder abuse is more prevalent among individuals with dementia or physical impairments. Fang et al. reported that 40% of elderly participants with cognitive or physical disabilities had experienced caregiver abuse or neglect, using data collected from geriatric and neurological clinics.^14^ Research conducted within the elderly community of Hong Kong reveals that over 60% of family caregivers admit to mistreating Alzheimer's patients. Elder abuse is frequently significantly underreported due to its concealment and the enduring impact of traditional values held by the elderly.^15^ However, no research has yet documented the prevalence or contributing factors of elder abuse among stroke survivors.

Several studies suggest a potential association between insomnia and an elevated risk of stroke.^16^ Chronic insomnia problems may correlate with risk factors for cardiovascular diseases, including hypertension, diabetes, and atherosclerosis, which are also prominent risk factors for stroke.^17^ Additionally, insomnia can cause decreased cerebral blood flow, leading to inadequate oxygen delivery to the brain and an elevated risk of stroke. Conversely, stroke can induce sleep disorders, manifesting as difficulties in falling asleep, frequent awakenings, and reduced sleep quality. Pathological alterations in the brain following a stroke, along with disturbances in neurotransmitters, can interfere with the regular regulation of sleep and potentially worsen insomnia symptoms. In summary, insomnia and stroke have a bidirectional relationship, potentially impacting each other in terms of pathogenesis and risk factors. Despite considerable research on post‐stroke insomnia, its prevalence remains notably elevated among stroke survivors. However, there is currently a lack of reports on the potential association between elder abuse among stroke survivors and the occurrence of insomnia.

This study aims to ascertain the prevalence and potential risk factors of elder abuse in stroke survivors, while also examining its correlation with insomnia through face‐to‐face interviews. By identifying groups at high risk of elder abuse, the study intends to provide valuable information for screening, intervention, and prevention efforts related to elder abuse in stroke patients. Ultimately, this research aims to improve the quality of life for stroke survivors and promote healthy aging

## 2 METHODS

### 2.1 Research population

This cross‐sectional study involved 485 patients (218 women and 267 men) aged 65−93 (average aged 74.2 ± 6.3), who sought stroke treatment at Shunde Hospital, Southern Medical University between January 2021 and June 2022 (Table 1). The investigators, consisting of medical personnel with expertise in epidemiology and health statistics, underwent rigorous and standardized training. Each participant was individually interviewed in a private room, ensuring strict confidentiality. To improve the participation rate of the elderly, health consultations and free physical examinations were offered before conducting the questionnaire. The questionnaire survey was conducted after obtaining oral “informed consent” from each elderly participant. For patients with severe cognitive and physical impairments, caregivers completed the questionnaire and documented their relationship with the elderly individuals. The project received ethical approval from the Ethics Committee of Shunde Hospital, Southern Medical University (KYLS20230607).

**Table 1:** Characteristics of the participants.

Characteristics	*N* (%)
Age	
65−80	390 (80.41)
>80	95 (19.59)
Gender	
Male	267 (55.05)
Female	218 (44.95)
Marital status	
Single/widow/divorced	206 (42.47)
Married	279 (57.53)
Education level	
Below high school	358 (73.81)
High school and above	127 (26.19)
Native residence	
Urban	278 (57.32)
Rural	207 (42.68)
Residence	
At home	252 (51.96)
Nursing homes	233 (48.04)
Number of children	
One or none	133 (27.42)
Two and more	352 (72.58)
Personal annual income (RMB)	
<5000	347 (71.55)
≥5000	138 (28.45)

The study included individuals who met the following criteria: a history of ischemic stroke, age over 65 years, and providing informed consent to participate. Those with psychosis or a negative attitude toward the study were excluded.

### 2.2 Calculation of sample size

In this study, we determine the sample size by considering the proportion of elder abuse among dementia patients, which is approximately 50%. We applied a 95% confidence level and 5% margin of error. The formula used for sample size calculation is as follows.

$$n=\frac{\left(1.96)2\times 0.5\times (1-0.5\right)}{0.052}=384$$

Taking into account an adopted sample ratio of 1:1.3 and a nonresponse rate of 20%, the final sample size was determined to be 576. Out of the investigators who participated, a total of 485 individuals provided their cooperation, resulting in a response rate of 84.20%.

### 2.3 Investigations

In this study, we utilized the elder abuse questionnaire from the Third Survey of Women's Social Status in China to investigate the prevalence of elder abuse among stroke survivors. The questionnaire, which has been extensively utilized (Cronbach's *⍺* = 0.81), consists of several items related to elder abuse within the family context. Participants were asked to report whether their family members/caregivers engaged in specific behaviors over the past year. These behaviors include: (1) Long‐term refusal to visit, greet, or communicate with them; (2) Failure to provide basic living expenses or secretly misappropriating their money; (3) Neglecting their care when needed; (4) Insults or threatening them; (5) Physically assaulting them; (6) Providing an unstable residence; (7) Failing to adequately feed or provide proper nutrition; (8) Restricting their freedom to leave the house. Based on the mentioned criteria, behaviors 5 and 8 were categorized as physical abuse, behaviors 1 and 4 as emotional abuse, behavior 2 as financial abuse, and behaviors 3, 6, and 7 as neglect. A respondent who reported any of these eight behaviors was considered to have experienced abuse.

Based on relevant studies, we have identified 11 potential risk factors as independent variables. These variables include gender (male and female), age (≥80 and <80), education level (below high school and high school and above), native residence (rural and urban), marital status (married and other marital status), residence (nursing home and at home), personal annual income (greater than or less than 5000¥), caregiver relationship status (relatives or employees), number of children (1 or more), smoking (nonsmoking and smoking), and drinking (not‐drinking and drinking).

### 2.4 Assessment of insomnia

Insomnia was assessed using a questionnaire based on the criteria outlined in Diagnostic and Statistical Manual of Mental Disorders‐IV (DSM‐IV). The questionnaire consisted of three questions:

Q1: “During the past month, how frequently did you experience difficulty falling asleep?”

Q2: “During the past month, how frequently did you wake up multiple times after initially falling asleep, resulting in sleep loss?”

Q3: “During the past month, how often have you experienced early morning awakenings, resulting in sleep loss?”

Participants provided answers on a 5‐point scale, where 0 represented “*never*,” 1 represented “*rarely*,” 2 represented “*sometimes*,” 3 represented “*often*,” and 4 represented “*always*.”

### 2.5 Statistical analysis

Data input and analysis were conducted using SPSS 23.0 software. Descriptive statistics were presented using frequencies and percentages. The initial assessment focused on the prevalence of elder abuse and insomnia among stroke survivors. The association between sociodemographic characteristics of the elderly and the occurrence of abuse, as well as the relationship between elder abuse and insomnia, was examined using the *χ* ^2^ test. Additionally, a logistic regression on significant variables during bivariate analysis was constructed to evaluate the connection between independent variables and elder abuse. The analysis results were assessed at a 95% confidence interval (CI), with statistical significance set at a *p* value <0.05.

## 3 RESULTS

### 3.1 Prevalence of elder abuse among stroke survivors

The study identified a total of 302 cases of elder abuse among stroke survivors, resulting in a prevalence rate of 62.27%. Emotional abuse (138 cases) and neglect (117 cases) were more common than economic abuse (69 cases) and physical abuse (59 cases). Among the participants, 14.85% reported experiencing two or more types of abuse (Table 2).

**Table 2:** Prevalence of elder abuse among stroke survivors.

Elder abuse	Freguency (*n*)	Percentage (%)
Total	302	62.27
Neglect	117	24.12
Emotional	138	28.45
Physical	69	14.23
Financial	59	12.16
≥2 types	72	14.85

### 3.2 Factors associated with elder abuse among stroke survivors

The study found that the prevalence of elder abuse was higher in the 80‐year‐old and above group (67.37%) compared to the 65‐ to 79‐year‐old group (61.03%). Women (65.60%) were more likely to be abused than men (59.55%). Divorced, widowed, and single individuals (68.44%) were more vulnerable to to be abused than married individuals (57.71%). In turn, the level of education significantly affects the occurrence of abuse (65.92% vs. 51.97%). Higher prevalence of elder abuse was observed in people from rural areas (67.63% vs. 58.27%), residing in nursing homes (69.10% vs. 55.95%), with lower incomes (70.32% vs. 42.03%), with one or none children (66.92% vs. 60.51%). The *χ* ^2^ test revealed a statistically significant difference in the prevalence of abuse among the various groups (*p* < 0.05) as indicated in Table 3.

**Table 3:** The relationship between social demographic characteristics and elder abuse.

	Total (*n*)	Abuse, *n* (%)	*χ* ^2^	*p*
Age			14.09	0.00
65−80	390	238 (61.03%)		
>80	95	64 (67.37%)		
Gender			4.49	0.03
Male	267	159 (59.55%)		
Female	218	143 (65.60%)		
Marital status			5.82	0.02
Single/widow/divorced	206	141 (68.44%)		
Married	279	161 (57.71%)		
Education level			7.77	0.01
Junior high school or lower	358	236 (65.92%)		
High school and above	127	66 (51.97%)		
Residence			4.42	0.04
Urban	278	162 (58.27%)		
Rural	207	140 (67.63%)		
Current address			8.90	0.00
At home	252	141 (55.95%)		
Sheltered homes	233	161 (69.10%)		
Number of children			1.6859	0.19
One or none	133	89 (66.92%)		
Two and more	352	213 (60.51%)		
Personal annual income (RMB)			33.6277	6.67e−9
<5000	347	244 (70.32%)		
≥5000	138	58 (42.03%)		

In addition, the elderly who smoked (73.99% vs. 55.77%), drank (74.67% vs. 56.72%), and cared by employees (67.67% vs. 57.31%) had a higher risk of abuse. The prevalence of elder abuse reported by the elderly themselves is higher than that reported by caregivers (65.28% vs. 54.92%) (Table 4).

**Table 4:** Drug use and elder abuse of stroke survivors.

	Total (*n*)	Abuse, *n* (%)		
Tobacco use			15.72	0.00
Yes	173	128 (73.99%)		
No	312	174 (55.77%)		
Alcohol use			14.21	0.00
Yes	150	112 (74.67%)		
No	335	190 (56.72%)		
Caregiver			5.53	0.02
Family members or relatives	253	145 (57.31%)		
Employees	232	157 (67.67%)		
Investigation filling personnel			4.18	0.04
The elderly	360	235 (65.28%)		
employee	122	67 (54.92%)		

Logistic regression analysis revealed that certain factors were associated with the likelihood of elder abuse. Elder individuals who were married (OR: 0.24, 95% CI: [0.15−0.38]), had higher education levels (OR: 0.33, 95% CI: [0.12−0.94]), and higher annual income (OR: 0.00, 95% CI: [0.00−0.02]) were found to be less likely to experience abuse. On the other hand, individuals residing in nursing homes (OR: 107.45, 95% CI: [22.18−520.45]) and smokers (OR: 22.60, 95% CI: [8.02−63.72]) were at a higher risk of elder abuse (Table 5).

**Table 5:** Logistic regression analysis of factors associated with elder abuse.

	Items	SE	*z* ☐	*p*	OR	95% CI
Elder abuse	Marital status (reference: Single/widow/divorced)	0.25	−5.82	0.00	0.24	0.15−0.38
	Education (reference: junior high school or lower)	0.53	−2.08	0.04	0.33	0.12−0.94
	Current address (reference: at home)	0.81	5.81	0.00	107.44	22.18−520.45
	Smoking (reference: nonsmoking)	0.53	5.89	0.00	22.60	8.02−63.72
	Annual income (reference: <5000 RMB)	0.84	−6.90	0.00	0.00	0.00−0.02
Neglect	Age (reference: aged 65−79)	0.37	5.60	0.00	7.88	3.82−16.22
	Education (reference: junior high school or lower)	0.56	−2.71	0.01	0.22	0.07−0.66
	Current address (reference: at home)	0.38	2.91	0.00	3.01	1.43−6.34
	Smoking (reference: nonsmoking)	0.37	5.43	0.00	7.26	3.55−14.84
	Number of children (reference: one or none)	0.59	−2.40	0.02	0.24	0.08−0.77
	Annual income (reference: <5000 RMB)	0.53	−3.11	0.00	0.20	0.07−0.55
	Investigation filling (reference: elder)	0.52	−2.05	0.04	0.35	0.13−0.96
Financial abuse	Age (reference: aged 65−79)	0.49	3.52	0.00	5.52	2.14−14.29
	Current address (reference: at home)	0.44	4.20	0.00	6.41	2.69−15.25
	Smoking (reference: nonsmoking)	0.43	2.65	0.01	3.13	1.35−7.27
	Annual income (reference: <5000 RMB)	0.43	−1.91	0.06	0.44	0.19−1.02
	Investigation filling (reference: elder)	0.53	−2.43	0.02	0.28	0.10−0.78
Emotional abuse	Gender (reference: female)	0.23	2.14	0.03	1.65	1.04−2.62
	Age (reference: aged 65−79)	0.48	6.28	0.00	20.45	7.97−52.45
	Current address (reference: at home)	0.40	3.61	0.00	4.21	1.93−9.17
	Smoking (reference: nonsmoking)	0.42	6.15	0.00	13.27	5.82−30.26
	Number of children (reference: one or none)	0.43	−2.24	0.03	0.38	0.16−0.89
	Annual income (reference: <5000 RMB)	0.47	−3.76	0.00	0.17	0.07−0.43
	Investigation filling (reference: elder)	0.48	−2.32	0.02	0.33	0.13−0.84
Physical abuse	Age (reference: aged 65−79)	0.45	3.43	0.00	4.71	1.94−11.40
	Current address (reference: at home)	0.41	3.30	0.00	3.84	1.73−8.53
	Smokin (reference: nonsmoking)	0.41	5.03	0.00	7.66	3.47−16.92
	Annual income (reference: <5000 RMB)	0.45	−2.94	0.00	0.27	0.11−0.65
	Investigation filling (reference: elder)	0.50	−2.81	0.01	0.24	0.09−0.65

Specifically, people over 80 years of age, living in nursing homes, with lower annual income, and who smoke were statistically more prone to neglect, financial abuse, emotional abuse, and physical abuse. Moreover, women were more likely to experience emotional abuse compared to men (OR: 1.65, 95% CI: [1.04−2.62]). Neglect was less frequently observed in individuals with higher education levels (OR: 0.22, 95% CI: [0.07−0.66]), while emotional abuse was less common in individuals with two or more children (OR: 0.38, 95% CI: [0.16−0.89]). Furthermore, employees reported neglect (OR: 0.35, 95% CI: [0.13−0.96]), financial abuse (OR: 0.28, 95% CI: [0.10−0.78]), emotional abuse (OR: 0.33, 95% CI: [0.13−0.84]), and physical abuse (OR: 0.24, 95% CI: [0.09−0.65]) less frequently compared to the elder themselves.

### 3.3 Prevalence of insomnia among stroke survivors and its association with elder abuse

Insomnia is a common issue among stroke patients and is known to have a significant impact on their health. However, there is a lack of research on the relationship between insomnia and elder abuse. In our study, we found that 51.34% of stroke survivors reported experiencing at least one sleep disorder. Specifically, 43.30% had trouble falling asleep, 41.65% struggled to maintain sleep, and 42.62% frequently woke up early. Among these individuals, 42.06% expressed dissatisfaction with their sleep quality (Supporting Information: Table S1).

We conducted an examination to explore the connection between elder abuse and insomnia. The results showed that stroke survivors who experienced elder abuse were more likely to have difficulties in maintaining sleep (*χ* ^2^ = 6.3095, *p* = 0.01) and early morning awakenings (*χ* ^2^ = 11.8194, *p* = 0.00). Additionally, patients who experienced elder abuse reported lower satisfaction with their sleep compared to those who did not (*χ* ^2^ = 7.0311, *p* = 0.01). However, there was no statistically significant difference in the overall prevalence of insomnia and difficulty falling asleep between the two groups (Table 6).

**Table 6:** Insomnia and elder abuse of stroke survivors.

		Elder abuse	Without elder abuse	*χ* ^2^	*p*
Insomnia	Yes	163	86		
	No	139	95	2.22	0.14
Difficulty initiating sleep	Yes	133	67		
	No	169	116	2.59	0.11
Difficulty maintaining sleep	Yes	139	63		
	No	163	120	6.31	0.01
Early morning awakening	Yes	154	68		
	No	148	115	11.82	0.00
Sleep satisfaction	Yes	161	120		
	No	141	63	7.03	0.01

## 4 DISSCUSSION

This study aims to assess the prevalence and risk factors of elder abuse among stroke survivors. By determining the severity of elder abuse among stroke survivors and identifying the associated risk factors, along with investigating the correlation between elder abuse and insomnia, effective policy changes and intervention measures can be developed to minimize elder abuse and improve the survival and quality of life of stroke survivors. Previous studies on chronic diseases and Alzheimer's disease have indicated that elder abuse and insomnia are frequently observed. However, this study is the first to demonstrate that elder abuse exacerbates the prevalence of insomnia and reduces sleep satisfaction among stroke survivors. Additionally, emotional abuse and neglect are the most prevalent subtypes of elder abuse among stroke survivors, followed by economic abuse and physical abuse. The most common risk factors associated with elder abuse among stroke survivors include income, smoking, and residing in a nursing home.

Elder abuse is a significant issue affecting elderly individuals in China who suffer from physical and cognitive disabilities. In this study, it was found that 62% of stroke survivors who participated reported experiencing at least one form of abuse.^18^ This prevalence rate is similar to that observed among other older adults in China with cognitive and physical impairments. For example, Yan et al. discovered a 62% prevalence of elder abuse in the past month among 122 family caregivers of elderly individuals with dementia, which was higher than the 42.8% prevalence of physical or psychological abuse reported by Fang et al. among 1002 elderly patients over the age of 55 with cognitive or physical disabilities.^19^ Moreover, there are variations in the prevalence of different types of abuse, with emotional abuse and neglect being the most prevalent, while physical and economic abuse rates are comparatively lower, consistent with previous studies. Additionally, more than 14% of patients reported experiencing multiple types of abuse, indicating that stroke survivors may be at risk of abuse from multiple sources in a caregiving context. Despite the high prevalence of elder abuse in China, it has not received sufficient attention.

Further analysis of risk factors confirmed that the prevalence of elder abuse is associated with age, educational background, residential address, smoking, income, and questionnaire responses. These findings support previous observations that higher income, higher education, younger age, and nonsmoking status significantly reduce the risk of elder abuse. Additionally, elder abuse is found in both community and institutional settings. Our research reveals a significantly increased risk of elder abuse among stroke survivors in nursing homes. Although our reported rate is lower than the 81% reported by Khulood Alraddadi among 446 participants in 2020, it is still higher than the rates in Macau (11.48%) and Guangzhou (8.24%).^20^, ^21^ It is worth noting that while the regression analysis identified nurses as protective factors for the prevalence of the four types of elder abuse, this may not be actually the case. The reported prevalence of elder abuse by nurses is lower than the actual situation, suggesting that nurses may not fully recognize certain behaviors that can cause distress to the elderly.

Numerous studies have demonstrated the association between elder abuse and the occurrence of various diseases, such as cardiovascular diseases, chronic obstructive pulmonary disease, digestive system diseases, metabolic diseases, and tumors.^22^ These diseases, in turn, contribute to physical and cognitive impairment, which further increases the risk of elder abuse. The mistreatment of older adults has significant medical implications, often affecting multiple physiological systems. However, despite insomnia being a common mental health condition among stroke patients, there have been few studies examining the link between elder abuse and sleep disorders.^23^ In our study, we found that 51.3% of the participants experienced sleep disorders, which aligns with previous reports of insomnia rates ranging from 30% to 68% among stroke survivors.^24^, ^25^ Insomnia is also considered a potential risk factor for physical disability, dementia, and stroke recurrence. Therefore, our research confirms that elder abuse is associated with an increased risk of insomnia, perpetuating a detrimental cycle involving insomnia, stroke, and elder abuse. Moreover, it is important to pay particular attention to the sleep maintenance difficulties, early morning awakenings, and sleep satisfaction of individuals who are vulnerable to elder abuse.

However, it is crucial to acknowledge the limitations of this study. First, the data collected relied on self‐reports from patients, which may be influenced by memory bias. Additionally, the lack of objective and quantifiable indicators for elder abuse could result in either underestimation or overestimation of its occurrence. For instance, elderly individuals may choose not to disclose abuse due to hesitancy, fear, or embarrassment. Lastly, due to time constraints during interviews and patient cooperation, we were unable to include all potential risk factors in our research, such as previous experiences of victimization.

## 5 CONCLUSION

Elder abuse is a significant issue among elderly individuals who suffered a stroke, with emotional abuse and neglect being the most frequently observed subtypes. Nursing homes and smoking are identified as the primary risk factors associated with elder abuse. Additionally, elder abuse contributes to an higher prevalence of insomnia among stroke survivors. There is an urgent need for prospective research to evaluate the effectiveness of various interventions aimed at safeguarding older adults and reducing abuse among stroke survivors.

## AUTHOR CONTRIBUTIONS

**Guifen Zhang**: Conceptualization; data curation; investigation; methodology; software; writing—original draft. **Minyuan Chen**: Data curation; investigation; writing—review and editing. **Peiyi Xiao**: Formal analysis; investigation. **Zhibo Peng**: Conceptualization; formal analysis; investigation; methodology; project administration; resources; visualization.

## CONFLICT OF INTEREST STATEMENT

The authors declare no conflict of interest.

## TRANSPARENCY STATEMENT

The lead author Zhibo Peng affirms that this manuscript is an honest, accurate, and transparent account of the study being reported; that no important aspects of the study have been omitted; and that any discrepancies from the study as planned (and, if relevant, registered) have been explained.

## Supporting information

Supporting information.

## Data Availability

The original contributions presented in this study are included in the article/Supplementary material, further inquiries can be directed to the corresponding author's.
