# Supplementary material for: Prevalence and risk factors of elder abuse in survivors of stroke: A cross‐sectional study
Source: Health Sci Rep. 2023 Oct 13;6(10):e1616. doi: 10.1002/hsr2.1616 (PMC10571011; doi:10.1002/hsr2.1616)
Supplement: Supplementary file 1 — Supporting information. [file HSR2-6-e1616-s001.docx]

Table S1 Prevalence of insomnia among stroke survivors

| Insomnia | Freguency（n） | Percentage(%) |
| --- | --- | --- |
| Total | 249 | 51.34 |
| Difficulty in falling asleep | 210 | 43.30 |
| Difficulty in maintaining sleep | 202 | 41.65 |
| Wake up early | 222 | 42.62 |
| Dissatisfaction | 204 | 42.06 |
